# Classification of octet AB-type binary compounds using dynamical charges: A materials informatics perspective

**DOI:** 10.1038/srep17504

**Published:** 2015-12-03

**Authors:** G. Pilania, J. E. Gubernatis, T. Lookman

**Affiliations:** 1 Materials Science and Technology Division, Los Alamos National Laboratory, Los Alamos 87545, NM, USA; 2 Theoretical Division, Los Alamos National Laboratory, Los Alamos 87545, NM, USA

## Abstract

The role of dynamical (or Born effective) charges in classification of octet AB-type binary compounds between four-fold (zincblende/wurtzite crystal structures) and six-fold (rocksalt crystal structure) coordinated systems is discussed. We show that the difference in the dynamical charges of the fourfold and sixfold coordinated structures, in combination with Harrison’s polarity, serves as an excellent feature to classify the coordination of 82 *sp*–bonded binary octet compounds. We use a support vector machine classifier to estimate the average classification accuracy and the associated variance in our model where a decision boundary is learned in a supervised manner. Finally, we compare the out-of-sample classification accuracy achieved by our feature pair with those reported previously.

Understanding the relationship between the chemical composition and the crystal structure of compounds is a classical problem in materials science. For many years it was argued that perhaps it is not possible to predict the crystal structure of most solids without sophisticated quantum mechanical computations. The reason was that such predictions require accurately distinguishing between two closely related structures whose energy differences are often less than 0.1% of the cohesive energy and often less than ~0.001% of the total energy per formula unit. However, use of informatics methods provides an alternative, principled avenue to tackle this challenging problem. With informatics, one attempts to identify relevant features (sometime referred to as descriptors) of materials which can lead to simple rules for classification by efficiently capturing the regularities or trends in the Periodic Table as well as the essential physics.

Recently, we studied the classification of the octet AB solids using informatics methods^1^. By using the ground state excess Born charge of the A atom in conjunction with a standard feature, we accurately classified these solids into six-fold co-ordinated (*i*.*e*., in rocksalt) and four-fold co-ordinated (*i*.*e*., in zincblende and wurtzite) crystal structures in a supervised machine learning task^1^. Our proposed scheme can readily be employed to predict coordination (four-fold *v*/*s* six-fold) of octet binary compounds if reliable experimental measurements of the excess Born charges are available. However, in the absence of measurements, theoretical computation of the excess Born charges requires *a priori* knowledge of the crystal structure of the compound (the four-fold or six-fold coordination for which Born charges need to be computed). This requirement for prior knowledge prevents our proposed classification scheme from being an efficient prediction scheme, that is, a scheme from which we can predict the crystal structure for a proposed octet of unknown crystal structure using a purely theoretical framework. Our present work demonstrates that this limitation can easily be overcome and the Born effective charges can be part of an unbiased predictive scheme if we consider the difference of the Born charges in the fourfold and sixfold coordinated octets as a feature.

Similar to past efforts^2^,^3^,^4^,^5^,^6^,^7^,^8^,^9^,^10^,^11^,^12^, our work here on the octets focuses on identification of effective features to classify or predict crystal structures in the octets. The first such scheme was proposed by Mooser and Pearson^2^. Their pair of chemical features of Pauling’s classical electronegativity scale and the average principle quantum number 
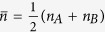
 was tremendously successful (with about 90% accuracy) in separating the fourfold and sixfold coordinated octet compounds (*cf*. Fig. 1a).

Next, Phillips and Van Vechten introduced two fully quantum-mechanical coordinates given by the average covalent energy gap *E*_*h*_ and the average ionic energy gap *C*, which separated exactly the fourfold (*i.e*., in zincblende (ZB) or wurtzite (WZ) crystal structures) and sixfold (*i.e*., in rocksalt (RS) crystal structure) coordinated octet binary compounds as shown in Fig. 1b 3,4,5. A third classification scheme was developed by St. John and Bloch^6^ based upon appropriate combinations of pseudo-potential orbital radii (termed as *r*_*σ*_ and *r*_*π*_) as determined by a Pauli-force model potential developed by Simons and Bloch (Fig. 1c)^13^,^14^,^15^,^16^,^17^. The feature pair used by them was extended to suboctet binary crystals by Chelikowsky *et al*.^7^ and Zunger^8^,^9^.

Finally, another chemical scale *χ*, characterizing each atom in the periodic table, was proposed by Pettifor^10^, which allows a single two-dimensional structure map (*χ*_*a*_, *χ*_*b*_) to be plotted for all binary compounds with a given stoichiometry AB_*n*_. Pettifor’s chemical scale *χ*, further evolved into *M*, the Pettifor’s Mendeleev number^11^,^12^. This number also allows a single two-dimensional structure plot for all AB solids. Each point in the plot is the pair (*M*_*A*_, *M*_*B*_). Although empirical, Pettifor’s more general scale separates octet as well as non-octet AB solids structurally and separates fourfold and sixfold coordinated octet sp bonded solids almost perfectly, as can be seen from Fig. 1e.

Recently, several groups revisited the problem of classification of the fourfold and sixfold coordinated octet AB *sp*–bonded compounds from a statistical learning perspective. In this context, the St. John-Bloch feature pair was used by Saad *et al*.^18^ as part of a larger feature set. Ghiringhelli and co-workers^19^ presented a general computational strategy to design physically meaningful features from a set of atom-derived properties. Their scheme identified *D*_1_ = [*IP*(*B*) − *EA*(*B*)]/*r*_*p*_(*A*)^2^ and *D*_2_ = |*r*_*s*_(*A*) − *r*_*p*_(*B*)|/exp(*r*_*s*_(*A*)) as an optimal feature pair for predicting the energy difference between RS and ZB crystal structures of a given AB octet compound. Here *IP*(*E*), *EA*(*E*), *r*_*s*_(*E*) and *r*_*p*_(*E*) for atom *E* represent the atomic ionization potential and electron affinity and the orbital radii (where the radial probability density of the valence *s* and *p* orbitals are maximal), respectively. The separation between the fourfold and sixfold binary compounds reached by this feature pair is depicted in Fig. 1f.

Previously we demonstrated that the feature pair with excess Born effective charge and St. John Bloch radii *r*_*σ*_ classified the six- and four-fold structures with an average success of 97% as evaluated on a 10%-leave-out cross validation test set^1^. Here we propose a more relevant feature pair (from a prediction point of view in a purely theoretical framework) *viz*. the difference of the Born charges in the fourfold and sixfold coordinated octets (ΔZ^*^) and Harrison’s bond polarity (*α*_*p*_). In what follows, we demonstrate that this feature pair works remarkably well in separating compounds with rocksalt and non-rocksalt crystal structures in a supervised machine learning task. We also compare the performance of our proposed feature pair with the other classifiers presented in Fig. 1 to find that it performs better than most of the feature pairs reported previously and almost as well as our previously proposed pair. Furthermore, for each pair, our analysis critically assesses the associated model variability in an automated machine learning classification task by providing rigorous confidence intervals to the average classification accuracy. We note that we compute *α*_*p*_ in the RS crystal structure for all the compounds considered in the present study and show therefore that it is an unbiased feature towards the aimed prediction task.

## Results

The binary compound semiconductors are known to crystallize in zincblende (ZB), wurtzite (WZ), rocksalt (RS), cesium chloride (CsCl), and diamond cubic (DC) crystal structures, with RS being the most common structure. These crystal structures also separate the solids into those that are strongly ionic bonded (for instance, CsCl) from those that have a mix of ionic and covalent bonding (for instance, RS, ZB and WZ systems) up to those that are purely covalent (DC systems). Out of the 82 *sp*–bonded binary octet compounds considered in the present study, only three adopt CsCl crystal structure and these sit near but apart from the rocksalts on any 2D classification map. Similarly, the four DC systems from the group IV of the periodic table, which are purely covalent, cluster near but apart from the wurtzites. Hence, it is relatively easy to classify CsCl and DC systems. The difficult task is drawing the boundary between the systems with RS, WZ and ZB crystal structures. Therefore, here we attempt to classify a system as RS or ZB/WZ. In other words, out of the three most prominent crystal structures exhibited by binary compound semiconductors (*viz*., RS, WZ and ZB), we would like to learn a classification scheme that separates the fourfold (ZB/WZ) and the sixfold (RS) coordinated compounds. For classification purposes only, the systems with CsCl and RS ground states were designated as rocksalts and the remaining systems with the other three crystal structures were labeled as non-rocksalts. Although our grouping is a simplification that reduces the number of boundaries machine learning needs to draw, it retains the core difficulty of the classification problem while enabling the use of well-developed machine learning methods for binary classification. Table I of the supplementary information presents further details of the 82 binary AB systems considered in the present study.

Our new feature pair, based on Born effective charges and bond polarity, is a quantification of dynamical and static polarizabilities of a material. The Born effective charge Z^*^ controls the long-range Coulomb part of the force constants and therefore is a fundamental quantity for the study of lattice dynamics. Formally, Z^*^ can be represented as the atomic position derivative of the polarization *P* at zero macroscopic electric field or as the linear-order coefficient between the electric field *E* and the force *F*_*j*_ which the field exerts on *j*^*th*^ ion (where derivative is calculated at zero atomic displacement) or alternatively as the mixed second derivative of the total energy Ω^*tot*^ with respect to both electric field and atomic displacement,


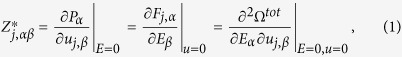


where *j* runs over the atoms, and *α* and *β* are Cartesian indices.

For each of the 82 binary AB systems, we computed Z^*^ in rocksalt as well as in a non-rocksalt (*i*.*e*., fourfold coordinated) crystal structure. For the calculation of Z^*^ in a fourfold coordinated environment, we choose the ZB crystal structure since in this structure the Z^*^ charge tensors for A and B atoms are diagonal with all three components equal (as is the case for the RS crystal structure). We note, however, that we could have used the WZ crystal structure instead of the ZB crystal structure for the computation of Z^*^ in a fourfold coordinated environment, since the Z^*^ tensor components in these two fourfold coordinated structures appear to be linearly correlated, as shown in the supplementary information. For both the RS and ZB crystal structures an eight-atom cubic supercell was used (*cf*., Fig. 2a) with its axes chosen such that the computed Born effective charge tensors for A and B atoms were diagonal with all three components equal. Figure 2b shows a parity plot that compares our calculated values of the Born effective charges with the corresponding experimental values^20^,^21^ for a number of binary systems that exist in the RS and ZB crystal structures. We see from the figure that, in general, good agreement exists between the experimentally reported values and our LDA computed values. The full list of the DFT-computed properties for the 82 binary AB materials can be found in the supplementary information. Further technical details on the computational methodology are provided in the Methods section of the manuscript.

Our second feature is Harrison’s polarity *α*_*p*_. Within the tight-binding formalism^20^ *α*_*p*_ is defined as 
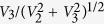
 and is a good measure of the polarity of a chemical bond. The covalent energy *V*_2_ is given by expression 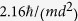, where *m* and *d* are mass of electron and the nearest neighbor (NN) distance between atoms in a given crystal, respectively. For *sp* bonded materials the polar energy *V*_3_ is defined as half of the absolute energy difference between the cation and anion *p* states, *i*.*e*., 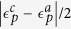. We used the values of 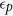 provided in Harrison’s Solid State Table^22^.

Our computed Z^*^ for the RS and ZB systems is displayed in Fig. 3(a,b). A striking feature of the figure is that Z^*^, for the systems with rocksalt ground state, computed in RS and ZB crystal structures (*cf*., Fig. 3a) does not differ much, whereas, a significant difference in the Z^*^ of RS and ZB crystals is exhibited by systems with a non-rocksalt ground state (Fig. 3b). In other words, we find that systems which prefer a four-fold coordination environment, in general, tend to show anomalous dynamical charges in a six-fold coordination environment, while the reverse is not true. This observation readily enables us to use the difference 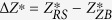 as a classification feature with RS systems exhibiting a smaller ΔZ^*^ and ZB/WZ systems falling on the other side of the spectrum with a relatively larger value of ΔZ^*^. Combining this feature with Harrison’s polarity, which is designed to naturally distinguish between polar and non-polar bonds, forms our 2D classifier presented in Fig. 3 (c).

From Fig. 3c we see that both ΔZ^*^ and *α*_*p*_ are good individual classifiers for the rocksalts and non-rocksalts. For the rocksalts, the *α*_*p*_ always takes values in the close vicinity of one while the ΔZ^*^ remains close to zero, owing to the near-equal values of 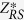 and 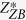 (Fig. 3a). This leads rocksalts to cluster in the top left corner of Fig. 3c, with a boundary clearly separating the rocksalts and non-rocksalts. However, we find that the compounds MgS and MgSe, which have been designated as RS in the literature, appear in our plot in the non-rocksalt domain. Experimentally, it is found that the free energy of the RS–ZB phase transition for these compounds is nearly zero^23^. Furthermore, these two systems were classified to be in the ZB structure in Zunger’s analysis^9^. Our DFT computations also reveal that the energy difference Δ*E* = *E*_*RS*_ − *E*_*ZB*_ for these systems is very small. An LDA calculation gives a Δ*E* of −0.088 eV/atom and −0.056 eV/atom for MgS and MgSe, respectively, while using the generalized gradient approximation (GGA) yields the values −0.015 eV/atom and 0.014 eV/atom, respectively.

**Figure 1:**
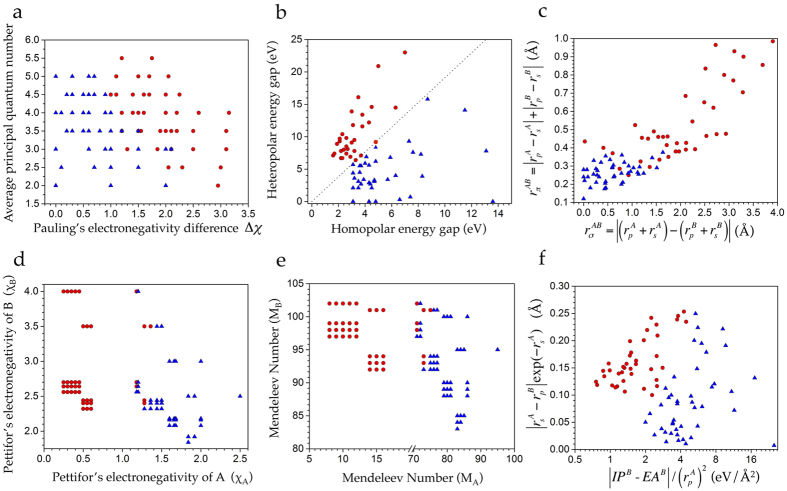
Various feature pairs previously proposed to classify AB–type *sp*–bonded compounds. (**a**) Mooser and Pearson’s (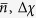)^2^, (**b**) Phillips and Van Vechten’s homo- and hetero-polar energy gaps (*E*_*h*_, *C*)^3^,^4^,^5^, (**c**) St. John and Bloch’s (*r*_*σ*_ and *r*_*π*_) pair based upon orbitally dependent radii^6^, (**d**) Pettifor’s chemical scale (*χ*_*A*_, *χ*_*B*_)^10^, (**e**) Pettifor’s Mandeleev number pair (*M*_*A*_, *M*_*B*_)^11^,^12^, and (**f**) Ghiringhelli *et al*.’s atom-derived feature pair (*D*_1_, *D*_2_)^19^. Orbital radii *r*_*s*_ and *r*_*p*_ used in panel (**c**) are those recently reported by Ghiringhelli *et al*.^19^ using the all-electron full-potential code FHI-aims^35^ and represent the radii at which the radial probability densities of the valence 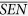 and *p* orbitals have their maxima. Red circles and blue triangles represent rocksalt and non-rocksalt classification labels. Each panel contains data for all 82 AB compounds studied here, except panel (**b**) where (*E*_*h*_, *C*) experimental data for only 69 of the 82 compounds was available^5^.

**Figure 2:**
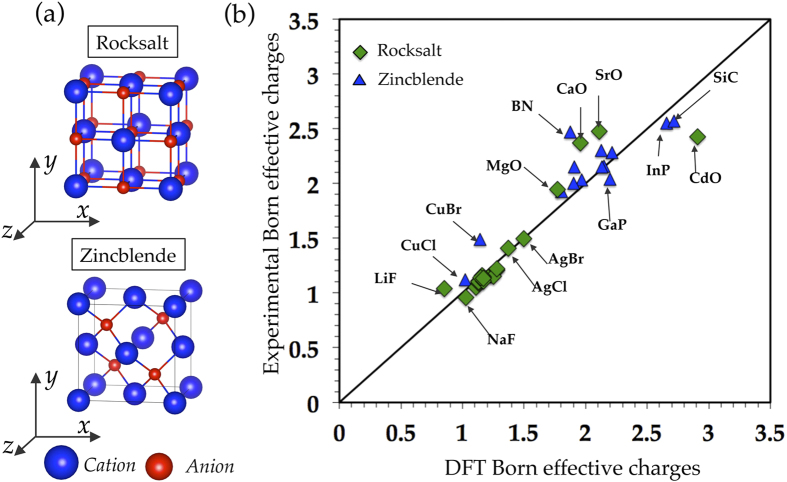
(**a**) RS (top) and ZB (bottom) supercells used in DFT computations to compute the Born effective charges. (**b**) Parity plot comparing our DFT computed Born effective cation charges with the corresponding experimental values^20^,^21^ for a number of AB binary octet compounds in RS and ZB crystal structures^36^,^37^,^38^.

**Figure 3:**
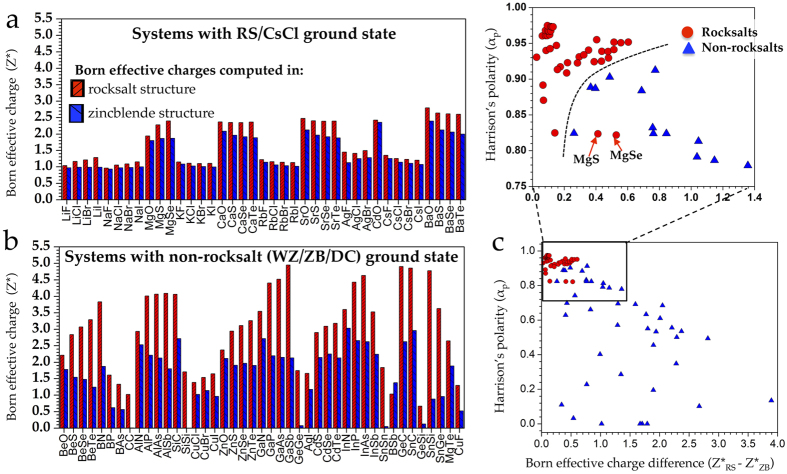
DFT computed Born effective charges for (a) systems with RS ground state (including three systems with CsCl crystal structures, namely, CsCl, CsBr and CsI) and for (b) systems with non-rocksalt ground state (including ZB, WZ and DC crystal structures). For each of the compounds Born effective charges are reported in the six-fold coordinated RS and four-fold coordinated ZB crystal structures. (**c**) Plot of our binary feature pair (ΔZ^*^, *α*_*p*_) that effectively separates the rocksalts and non-rocksalts.

Once we have a feature pair that efficiently classifies the materials under investigation, we can draw a curved decision boundary to separate the rocksalts from the non-rocksalts compounds on a 2D plot. Such a decision boundary is shown in Fig. 3c. Traditionally, this approach was used to classify the binary AB systems (for instance, in the features plotted in Fig. 1a–e). An alternative approach is to draw the decision boundary using well-developed machine learning (ML) techniques. ML-based techniques not only allow us to identify an optimal decision boundary in an automated manner, but also make it possible to quantitatively compare the classification accuracy between different feature pairs using well developed statistical techniques. Furthermore, since machine learning can handle any number of features, we can systematically try to improve the classification accuracy of a model by gradually adding more relevant features in the model.

In the present study, we used a support vector machine classifier^24^,^25^ to quantify the classification accuracy of our (ΔZ^*^, *α*_*p*_) feature pair and to compare it with the accuracy achieved by those discussed previously. We evenly split the data into a training and a test set and used a Gaussian radial basis function (RBF) kernel with its hyper-parameter optimized in an internal grid search 5-fold cross-validation loop on the training data^26^. We also note that taking 50% of the available data in the test set for out-of-sample error estimation is quite a stringent model assessment criterion. Further details on the machine learning methodology are provided in the methods section of the manuscript.

**Figure 4:**
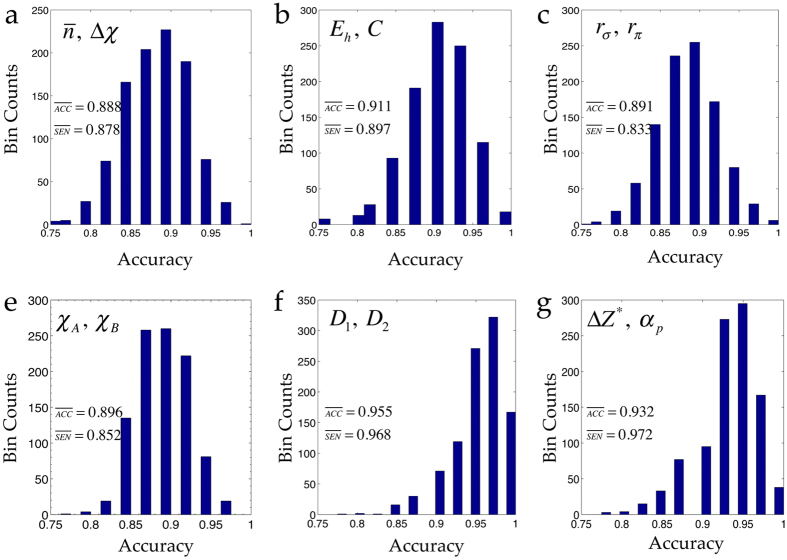
Histograms of correct rate of classification between the rocksalts and non-rocksalts achieved by different feature pairs in a support vector machine classifier analysis over 1000 separate runs. Average correct rate or accuracy 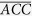 and average sensitivity 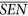 are also reported in each of the panels. The features are defined in caption of Fig. 1.

After fitting the model parameters to the training data, we evaluated the accuracy of the model with respect to how well it classifies the instances in the test data which were never seen by the trained model before. To quantify the classification accuracy we used *correct rate* and *sensitivity* as metrics. The correct rate is defined as the fraction of correctly classified samples in a randomly selected test set, while sensitivity represents the fraction of correctly classified non-rocksalts out of the total non-rocksalt samples in a randomly selected test set. To estimate the average classification accuracy and associated variance of a given model, we ran the support vector machine classifier analysis 1000 times for the feature pairs presented in Fig. 1, including our proposed descriptor pair (ΔZ^*^, *α*_*p*_). Histograms of correct rate of classification (or classification accuracy) achieved for each of these feature pair are reported in Fig. 4(a–g). Average correct rate or accuracy (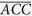) and average sensitivity (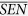) are also given in each the the panels. A comparison of 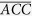 and 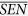 across the models shows that the (ΔZ^*^, *α*_*p*_) pair achieves the second best 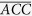 and the highest 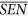.

Finally, we note that while the average accuracy might be reasonably estimated by averaging over the *k* folds of a *k*-fold cross validation, this average for small datasets in general has a large variance associated with it that is not estimated well by the variance computed with a *k*-fold cross-validation. This is a well known problem in studying small datasets that has also been noted in bioinformatics^27^.

## Discussion

Most previously reported feature pairs proposed for coordination classification of the octets (*cf*. Fig. 1) are formulated as a quantification of polarity or covalency of the chemical bond. For instance, *r*_*σ*_ in the St. John and Bloch^6^ pair is a measure of the electronegativity difference between the atoms A and B, whereas, *r*_*π*_ measures the average *sp*-orbital hybridization. In line with this classical notion, our feature pair, based on the Harrison’s bond polarity^22^ and the Born effective (or dynamical) charges, can be regarded as a convenient measure of static and dynamical bond polarizabilities. Since the learning task at hand requires one to distinguish between highly ionic rocksalt compounds from those which are partly ionic and partly covalent, the high classification accuracy achieved by the proposed descriptor pair can easily be rationalized.

We further note that the ΔZ^*^ and *α*_*p*_ are each good classifiers individually for separating the rocksalts from the non-rocksalt compounds. Since these two quantities are poorly correlated with each other, combining them in a 2D feature pair lends itself as an effective classifier. The same was also true for the excess Born charge, one of the components in our previously reported feature pair^1^. Using chemical intuition to search for features with this type of physical quality is an alternative to finding high throughput functional combinations of features that improve classification performance^19^.

**Figure 5:**
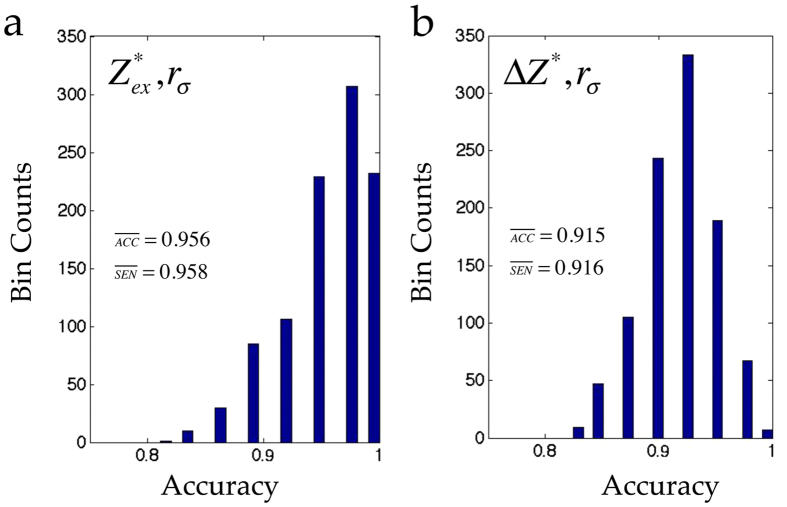
Histograms of correct rate of classification between the rocksalts and non-rocksalts achieved for 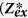, *r*_*σ*_) and (ΔZ^*^, *r*_*σ*_) in a support vector machine classifier analysis over 1000 separate runs. Average corect rate or accuracy 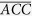 and average sensitivity 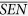 are also reported in each of the panels. 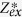 and ΔZ^*^ are the excess Born effective charge (defined as a difference between the Born effective charge and the nominal charge of a cation) and difference in the Born effective charge of RS and ZB crystals, respectively.

We discuss the classification performance of the pair (ΔZ^*^, *α*_*p*_) in comparison to our recently reported pair of excess Born effective charge 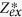 and *r*_*σ*_^1^. The 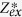 is defined as a difference between the Born effective charge and the nominal charge of a cation. In a machine learning run consistent with those mentioned above, the (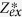, *r*_*σ*_) pair achieves a performance very close to that of the (ΔZ^*^, *α*_*p*_) pair with 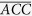 = 0.956 and 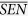 = 0.958 (*cf*. Fig. 5(a)). We do note however that in a purely theoretical framework the (ΔZ^*^, *α*_*p*_) pair is a more relevant coordination predictor than the (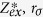) pair, since one does not have to know the stable crystal structure before hand to compute the 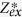. Nevertheless, as the classic Phillips and Van Vechten’s features, the 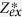 are measurable and therefore the (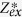, *r*_*σ*_) pair is still a valuable feature pair in a situation where the Born charges are directly accessible from experimental measurements. Finally, we note that pairing ΔZ^*^ with *r*_*σ*_ resulted in a slightly poor performance with 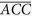 = 0.915 and 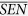 = 0.916, as compared to the (ΔZ^*^, *α*_*p*_) pair (*cf*. Fig. 5(b)).

Using the machine learning based classification approach allows us to go beyond the 2D feature pairs proposed in the past. We can try to systematically improve the classification model by combining more than two features. To demonstrate this, we present an example where we add Ghiringhelli *et al*.’s features from ref. 19 to our (ΔZ^*^, *α*_*p*_) feature pair. Learning with these four features and then making predictions on a randomly selected test set of 50% candidates in 1000 classification runs resulted in both improved 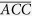 ( = 0.965) and 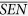 ( = 0.977). More significantly, this model resulted in a much smaller variance of 8.4 × 10^−4^ in the correct rate computed over the 1000 runs, as compared to the variances of 1.2 × 10^−3^ and 1.5 × 10^−3^ obtained with (*D*_1_, *D*_2_) or (ΔZ^*^, *α*_*p*_), respectively. This result clearly indicates that the classification model with the four features is consistently classifying better than the individual pairs when used separately.

## Conclusions

To conclude, we identified the Born effective charge as an excellent feature to classify octet AB-type binaries between four-fold (ZB/WZ crystal structures) and six-fold (RS crystal structure) coordinated compounds. We demonstrated that with the difference in the dynamical charges of the fourfold and sixfold coordinated structures, in combination with Harrison’s polarity, we can classify the coordination of *sp*–bonded 82 binary octet compounds to a much higher accuracy and confidence in an automated supervised learning task than most of the previously reported feature pairs. Furthermore, the proposed feature pair aligns very well with chemical intuition and can be interpreted as a measure of static and dynamical bond polarizabilities. We used the machine learning approach to evaluate and compare out-of-sample prediction performance of the proposed feature with those reported previously in the literature. Finally, we note that the statistical learning paradigm adopted here is not limited to a 2D feature pair and can be used to systematically combine more than two features to further improve the classification accuracy.

## Methods

### First principles computations

The NN distances (required to calculate *α*_*p*_ in the RS crystal structure) and the cation Z^*^ for the AB systems were computed using density functional theory (DFT) as implemented in the Vienna *ab initio* simulation package (VASP)^28^. For the exchange-correlation interaction^29^ the local-density approximation (LDA)^30^ was used. We note that the LDA provides a good description of the studied materials. The electronic wave functions are expanded in plane waves up to a cut-off energy of 500 eV. The pseudopotentials based on the projector augmented wave (PAW)^31^ method and Monkhorst-Pack sampling^32^ of the Brillouin-zone integrations were used. To obtain a geometry optimized equilibrium structure, atomic positions and the lattice parameters were fully relaxed using the conjugate gradient method until the stress components in all directions were less than 1.0 × 10^−3^ GPa. Density functional perturbation theory^33^,^34^ as implemented in VASP, was used to compute the Born effective charges.

### Machine learning details

In support vector machine binary classification, each instance of data is described by a vector of features 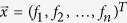 and a label *y*. In our case, the label *y* has a value of +1, for rocksalts, and −1, for non-rocksalt systems. A trained support vector machine finds a function that for any given 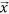 has a value of ±1, based on which out-of-sample predictions are made. Ideally, the machine learning process is aim at generating a decision boundary (or hypersurface) in the feature space that maximizes the distance of the closest instance from either class from it. This distance is also known as the margin^24^,^25^. Instances falling on the margin are called support vectors. In general a clear separation of the data via a finite margin is not possible so a soft margin support vector machine is constructed, which allows misclassification of instances (*i*.*e*., it allows points in the margin). If we represent our input data by the set of labeled instances 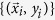, then a soft margin support vector classifier determines the hypersurface in the space of features by solving


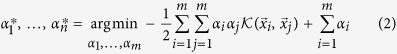


subject to constraints


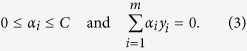


Adjusting *C* controls the number of misclassifications. In the minimization the competition is between the size of the margin and the degree of misclassification acceptable. The support vectors are now those 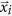 for which 0 < *α*_*i*_ < *C*. 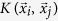 is called the kernel. In our case a Gaussian kernel with radial basis function, *i*.*e*., 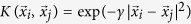, was used.

## Additional Information

**How to cite this article**: Pilania, G. *et al*. Classification of octet AB-type binary compounds using dynamical charges: A materials informatics perspective. *Sci. Rep*. **5**, 17504; doi: 10.1038/srep17504 (2015).

## Supplementary Material

Supplementary Information
