# Supplementary material for: Classification of octet AB-type binary compounds using dynamical charges: A materials informatics perspective
Source: Sci Rep. 2015 Dec 3;5:17504. doi: 10.1038/srep17504 (PMC4668360; doi:10.1038/srep17504)
Supplement: Supplementary Information [file srep17504-s1.pdf]

Supplementary Information for Classification of Octet AB-type binary compounds using Dynamical Charges: A Materials Informatics Perspective

G. Pilania, J. E. Gubernatis and T. Lookman*

E-mail: gpilania@lanl.gov

Table 1: Classification labels, experimentally observed crystal structures, nearest neighbor (NN) distances, Born effective charges (both in rocksalt and zincblende crystal structures) and polarity α_p for the AB binary alloys studied in the present study. NN distances and Born effective charges were calculated using density functional theory based computations within local density approximation (LDA). RS, ZB, W, G, DC and CsCl are used to denote rocksalt, zincblende, wurtzite, graphite, diamond cubic and cesium chloride crystals, respectively. An asterisk has been used for BSb and several group IV systems for which no experimental crystal structure is available.

Binary Alloy	Classification Label	Observed Structure	LDA NN distance in RS (Å)	Born effective charges in ZB (e)	Born effective charges in RS (e)	α_p
LiF	rocksalt	RS	1.953	0.97	1.04	0.892
LiCl	rocksalt	RS	2.481	0.99	1.16	0.917
LiBr	rocksalt	RS	2.655	0.99	1.21	0.923
LiI	rocksalt	RS	2.903	0.99	1.28	0.931
NaF	rocksalt	RS	2.219	0.94	0.96	0.931
NaCl	rocksalt	RS	2.715	0.97	1.06	0.940

Continued on next page

*To whom correspondence should be addressed

Table 1 – continued from previous page

Binary Alloy	Classification Label	Observed Structure	LDA NN distance in RS (Å)	Born effective charges		α_p
				in ZB (e)	in RS (e)	
NaBr	rocksalt	RS	2.880	0.98	1.09	0.943
NaI	rocksalt	RS	3.121	1.00	1.15	0.947
MgO	rocksalt	RS	2.076	1.80	1.94	0.825
MgS	rocksalt	RS/W	2.562	1.87	2.28	0.823
MgSe	rocksalt	RS	2.695	1.87	2.40	0.822
KF	rocksalt	RS	2.586	1.09	1.15	0.961
KCl	rocksalt	RS	3.043	1.03	1.11	0.961
KBr	rocksalt	RS	3.191	1.01	1.10	0.961
KI	rocksalt	RS	3.413	1.00	1.11	0.962
CaO	rocksalt	RS	2.360	2.09	2.37	0.922
CaS	rocksalt	RS	2.786	1.96	2.35	0.924
CaSe	rocksalt	RS	2.900	1.92	2.35	0.925
CaTe	rocksalt	RS	3.109	1.89	2.37	0.930
RbF	rocksalt	RS	2.734	1.14	1.22	0.968
RbCl	rocksalt	RS	3.192	1.06	1.16	0.967
RbBr	rocksalt	RS	3.337	1.04	1.14	0.967
RbI	rocksalt	RS	3.556	1.02	1.13	0.968
SrO	rocksalt	RS	2.539	2.12	2.48	0.940
SrS	rocksalt	RS	2.956	1.97	2.41	0.939
SrSe	rocksalt	RS	3.066	1.92	2.39	0.939
SrTe	rocksalt	RS	3.268	1.88	2.40	0.941
AgF	rocksalt	RS	2.399	1.13	1.45	0.934
AgCl	rocksalt	RS	2.683	1.25	1.41	0.913
AgBr	rocksalt	RS	2.797	1.28	1.50	0.909
CdO	rocksalt	RS	2.327	2.36	2.43	0.870
CsF	rocksalt	RS	2.910	1.26	1.35	0.975
CsCl	rocksalt	CsCl	3.373	1.14	1.26	0.973
CsBr	rocksalt	CsCl	3.517	1.11	1.23	0.973
CsI	rocksalt	CsCl	3.734	1.07	1.20	0.973
BaO	rocksalt	RS	2.740	2.39	2.80	0.955
BaS	rocksalt	RS	3.151	2.13	2.64	0.952
BaSe	rocksalt	RS	3.256	2.06	2.62	0.951

Continued on next page

Table 1 – continued from previous page

Binary Alloy	Classification Label	Observed Structure	LDA NN distance in RS (Å)	Born effective charges		α_p
				in ZB (e)	in RS (e)	
BaTe	rocksalt	RS	3.447	2.00	2.60	0.952
BeO	non-rocksalt	W	1.793	1.78	2.21	0.698
BeS	non-rocksalt	ZB	2.279	1.55	2.84	0.695
BeSe	non-rocksalt	ZB	2.418	1.48	3.07	0.692
BeTe	non-rocksalt	ZB	2.635	1.24	3.29	0.684
BN	non-rocksalt	ZB/W/G	1.730	1.88	3.83	0.402
BP	non-rocksalt	ZB	2.137	0.62	1.61	0.228
BAs	non-rocksalt	ZB	2.272	0.56	1.33	0.195
CC	non-rocksalt	DC/G	1.752	0.01	1.02	0.000
AlN	non-rocksalt	W	2.007	2.53	2.94	0.629
AlP	non-rocksalt	ZB	2.502	2.21	4.01	0.551
AlAs	non-rocksalt	ZB	2.608	2.13	4.07	0.533
AlSb	non-rocksalt	ZB	2.830	1.80	4.09	0.501
SiC	non-rocksalt	ZB/W	2.003	2.72	4.06	0.286
SiSi	non-rocksalt	DC	2.503	0.00	1.71	0.000
CuCl	non-rocksalt	ZB/W	2.467	1.02	1.39	0.889
CuBr	non-rocksalt	ZB/W	2.598	1.14	1.54	0.887
CuI	non-rocksalt	ZB	2.764	0.96	1.65	0.884
ZnO	non-rocksalt	W	2.111	2.11	2.38	0.824
ZnS	non-rocksalt	ZB	2.486	1.91	2.95	0.791
ZnSe	non-rocksalt	ZB/W	2.611	1.97	3.12	0.786
ZnTe	non-rocksalt	ZB	2.803	1.90	3.26	0.779
GaN	non-rocksalt	W	2.100	2.72	3.55	0.661
GaP	non-rocksalt	ZB	2.533	2.20	4.40	0.556
GaAs	non-rocksalt	ZB	2.634	2.15	4.52	0.536
GaSb	non-rocksalt	ZB	2.828	2.13	4.95	0.494
GeGe	non-rocksalt	DC	2.652	0.08	1.75	0.000
AgI	non-rocksalt	ZB/W	2.954	1.18	1.66	0.903
CdS	non-rocksalt	ZB/W	2.677	2.15	2.90	0.832
CdSe	non-rocksalt	W	2.789	2.25	3.10	0.824
CdTe	non-rocksalt	ZB	2.971	2.13	3.18	0.813
InN	non-rocksalt	W	2.318	3.03	3.60	0.742

Continued on next page

Table 1 – continued from previous page

Binary Alloy	Classification Label	Observed Structure	LDA NN distance in RS (Å)	Born effective charges		α_p
				in ZB (e)	in RS (e)	
InP	non-rocksalt	ZB	2.722	2.66	4.43	0.634
InAs	non-rocksalt	ZB	2.807	2.62	4.63	0.611
InSb	non-rocksalt	ZB	2.996	2.24	3.53	0.571
SnSn	non-rocksalt	DC	3.006	0.05	1.84	0.000
BSb	non-rocksalt	*	2.468	1.38	1.04	0.110
GeC	non-rocksalt	*	2.165	2.62	4.90	0.348
SnC	non-rocksalt	*	2.358	2.96	4.86	0.456
GeSi	non-rocksalt	*	2.579	0.13	0.67	0.032
SnSi	non-rocksalt	*	2.775	0.88	4.78	0.134
SnGe	non-rocksalt	*	2.832	0.96	3.63	0.102
MgTe	non-rocksalt	W	2.923	1.89	2.65	0.824
CuF	non-rocksalt	ZB	2.200	0.52	1.30	0.912

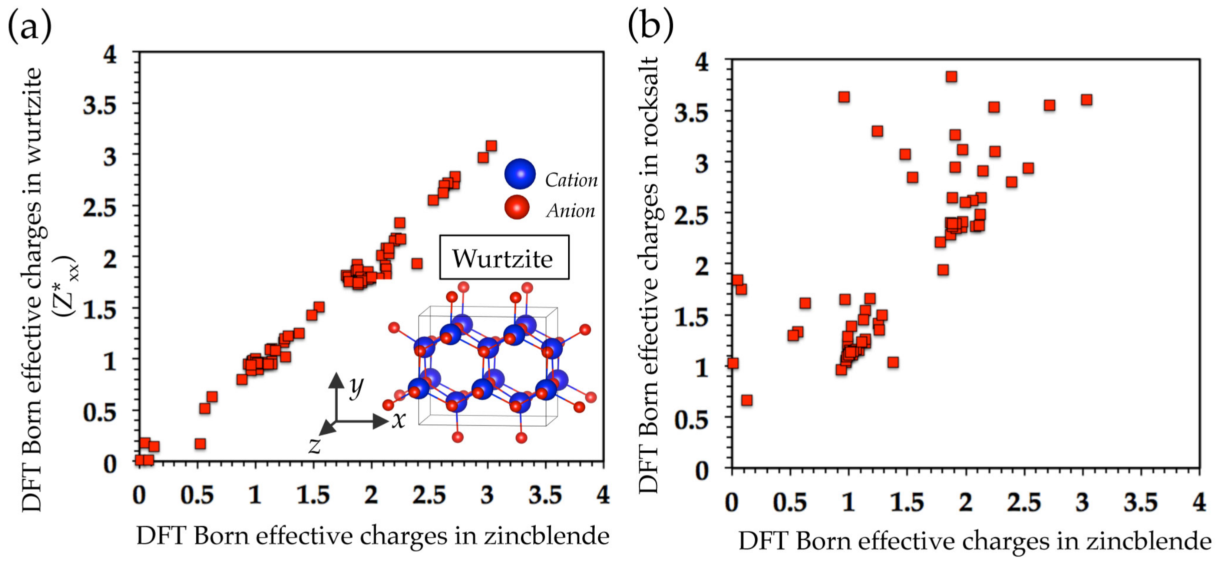

Figure 1: Parity plots comparing DFT computed Born effective charges of the octet AB binary alloys in zincblende crystal structure with those (a) in wurtzite and (b) in rocksalt crystal structures. For wurtzite crystal structure not all the components of the diagonal Born effective charge tensor are equal, unlike the two cubic crystals. Only xx component of the Born effective charge tensor of wurtzite is plotted on y axis in the panel (a). Supercell used for the wurtzite crystal structure is also shown in the panel.
